# Lactate Produced by Glycogenolysis in Astrocytes Regulates Memory Processing

**DOI:** 10.1371/journal.pone.0028427

**Published:** 2011-12-13

**Authors:** Lori A. Newman, Donna L. Korol, Paul E. Gold

**Affiliations:** 1 Neuroscience Program, University of Illinois at Urbana-Champaign, Champaign, Illinois, United States of America; 2 Institute for Genomic Biology, University of Illinois at Urbana-Champaign, Champaign, Illinois, United States of America; 3 Department of Psychology, University of Illinois at Urbana-Champaign, Champaign, Illinois, United States of America; 4 Department of Molecular and Integrative Physiology, University of Illinois at Urbana-Champaign, Champaign, Illinois, United States of America; 5 Department of Psychiatry, University of Illinois at Urbana-Champaign, Champaign, Illinois, United States of America; 6 Department of Biomedical Engineering, University of Illinois at Urbana-Champaign, Champaign, Illinois, United States of America; Georgia Health Sciences University, United States of America

## Abstract

When administered either systemically or centrally, glucose is a potent enhancer of memory processes. Measures of glucose levels in extracellular fluid in the rat hippocampus during memory tests reveal that these levels are dynamic, decreasing in response to memory tasks and loads; exogenous glucose blocks these decreases and enhances memory. The present experiments test the hypothesis that glucose enhancement of memory is mediated by glycogen storage and then metabolism to lactate in astrocytes, which provide lactate to neurons as an energy substrate. Sensitive bioprobes were used to measure brain glucose and lactate levels in 1-sec samples. Extracellular glucose decreased and lactate increased while rats performed a spatial working memory task. Intrahippocampal infusions of lactate enhanced memory in this task. In addition, pharmacological inhibition of astrocytic glycogenolysis impaired memory and this impairment was reversed by administration of lactate or glucose, both of which can provide lactate to neurons in the absence of glycogenolysis. Pharmacological block of the monocarboxylate transporter responsible for lactate uptake into neurons also impaired memory and this impairment was not reversed by either glucose or lactate. These findings support the view that astrocytes regulate memory formation by controlling the provision of lactate to support neuronal functions.

## Introduction

Glucose is an important modulator of memory in multiple tasks and species, with extensive work showing that peripheral and central administration of glucose enhances memory and that glucose may be an important mediator of epinephrine effects on memory [1], [2], [3], [4], [5], [6]. For example, glucose administered systemically to humans and rats significantly reverses age-related memory loss when given before or after information acquisition [7], [8], [9], [10] and improves memory in patients with Alzheimer's disease and Down's syndrome [11], [12]. Moreover, meals that create a slow, steady release of glucose improve memory in children and individuals who have better glucose tolerance perform better at memory tasks [10], [13], [14].

Although the adult brain relies heavily on glucose for its energy needs, the extracellular glucose levels are some of the lowest in the body, approximately 1 mM in the hippocampus vs. 5 mM in blood [15], [16], [17], [18]. In addition, extracellular glucose levels in the rat hippocampus during memory tests are dynamic, decreasing in response to memory tasks and loads [19], [20], [21]. In particular, when rats are tested for memory in a 4-arm spontaneous alternation maze, glucose levels decrease substantially; memory is enhanced by systemic glucose administration at doses that reverse that depletion as well as by microinjections of glucose directly into the hippocampus [19], [22]. These decreases in extracellular glucose levels in the hippocampus are not the result of locomotor activity or of alternation behavior *per se*: Rats tested on a 3-arm maze, an easier task for rats due to the lower working memory load of three versus four locations, make a similar number of arm entries. However, the rats exhibit only slight decreases in extracellular glucose levels and memory is not improved by glucose injections [21]. These findings suggest that basal glucose levels are sufficient for the energy demands of the easier task but not the harder one.

While the mechanism by which glucose acts on the brain to regulate memory is unclear, there is evidence that glucose augments training-related release of acetylcholine in the hippocampus [8], [22], [23], [24], an effect that may participate in glucose enhancement of memory. In addition, glucose effects on memory may include downstream effects mediated by the mammalian target of rapamycin (mTOR) pathway to promote mechanisms of neuronal plasticity [25]. mTOR is itself down-regulated by activation of the metabolic sensor, AMP-activated protein kinase (AMPK) [26], in response to cellular energy stress as might occur during training-associated decreases in glucose availability. Recent evidence suggests that the coordinated functions of mTOR and AMPK up- and down-regulate neuronal plasticity, respectively [27].

Neurons have two main sources of neural energy substrates, both beginning with circulating glucose. The first is glucose entry into neurons with subsequent oxidative metabolism. In addition to direct entry of glucose into neurons, a second source is provided by glucose entry into astrocytes. Unlike neurons, astrocytes store glycogen that can be rapidly metabolized upon activation to initiate glycogenolysis, thereby providing lactate as an energy substrate transported to neurons. Thus, astrocytic storage of glycogen provides a supplemental energy reserve available to neurons when demand is high [28], [29], [30], [31], [32].

While glucose uptake into neurons and astrocytes is about equal at baseline, recent findings show that whisker stimulation of somatosensory neocortical activity results in a preferential increase in glucose uptake into astrocytes [33]. Together, these findings lead to a general hypothesis that basal brain extracellular glucose levels can fulfill neuronal energy requirements under low-need conditions but, when the need is greater, for example during more intense cognitive functions, astrocytic glycogenolysis is activated to provide lactate, which is transported to neurons to provide a rapid boost from energy reserves when extracellular glucose levels are not sufficient to maintain optimal function.

The functional significance of this reserve for learning and memory is supported by recent findings that interference with lactate transport from astrocytes into neurons impairs long-term potentiation and long-term memory for an inhibitory avoidance task [32]. The transport is mediated by monocarboxylate transporters (MCTs) distinctly localized on astrocytes (MCT1 and MCT4), to release lactate, and neurons (MCT2) to admit lactate [34], [35], [36]. Because pretraining injections of a lactate transport inhibitor did not impair memory tested 1 hr after training, the authors concluded that astrocytic glycogenolysis was selectively necessary for long- but not short-term memory.

**Figure 1:**
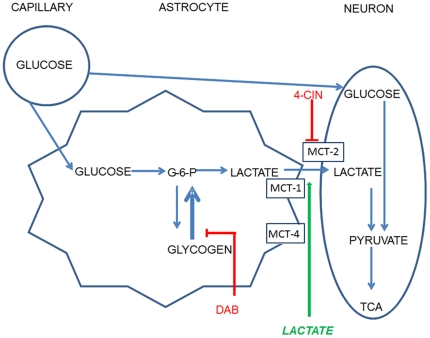
Model of astrocytic contribution of lactate to memory processing. Pharmacological tests and measures of many aspects of this figure were tested in the present experiments. DAB: 1,4-dideoxy = 1,4-imino-D-aribinitol, 4-CIN: α-cyano-4-hydroxycinnamate, MCT: monocarboxylate transporter.

The present experiments examine possible astrocytic involvement in a spontaneous alternation task, in which spatial working memory is assessed during short-term memory tests. Supporting the idea that memory in the spontaneous alternation task might be mediated by astrocytic glycogenolysis, previous findings indicate that systemic and central injections of glucose enhance memory in this task and reverse age-related memory impairments. Figure 1 illustrates the model tested here with pharmacological and neurochemical methods to evaluate the significance for memory of astrocytic glycogen metabolism to lactate and transport to neurons. In the present experiments, changes in extracellular lactate and glucose levels were assessed with bioprobes, enabling sampling every second. The roles of lactate and glucose in memory were further evaluated with selective pharmacological agents to block glycogenolysis [37] and MCT2 [38].

## Methods

### Subjects

Male, Sprague-Dawley rats (Harlan Laboratories; 3 months old at the time of surgery) were housed individually with free access to food and water and maintained on a 12∶12 hr light/dark cycle with lights on at 7:00 am. All procedures described in this paper were approved by the University of Illinois Urbana-Champaign Institutional Animal Care and Use Committee in accordance with guidelines outlined in *Guide for Care and Use of Laboratory Animals* and accredited by the Association for Assessment and Accreditation of Laboratory Animal Care.

### Surgeries

Rats were anesthetized with isoflurane and placed in a stereotaxic frame. In studies of the effects on memory of intrahippocampal drug infusions, two 8-mm guide cannulae (Plastics One) were chronically implanted bilaterally above the central portion of the ventral hippocampus (coordinates: 5.5 mm posterior and ±4.8 mm lateral from bregma and 4.2 mm ventral from skull) to accommodate infusion cannulae near the time of behavioral testing. Due to the shape and size of the housing for the wireless potentiostat used for measurements of changes in extracellular glucose and lactate levels during behavioral testing, the guide cannula for bioprobes needed to be placed close to the midline of the skull. Therefore the guide cannulae for bioprobes were implanted above the dorsal hippocampus instead of the ventral hippocampus in either the left or right hemisphere (coordinates: 3.8 mm posterior and 2.5 mm lateral from bregma and 1.9 mm ventral from skull). We have previously found that direct infusions of glucose into either the dorsal [39] or ventral [22], [24] hippocampus were both effective in enhancing memory during spontaneous alternation testing. All rats were allowed at least 1 week to recover after surgery during which rats were handled 3 min each day for 5 consecutive days prior to behavioral testing.

### Memory testing

Spatial working memory was assessed using spontaneous alternation tasks [cf: 40], [41], [42]. Spontaneous alternation was chosen to assess memory because it requires no food reward and thus no food restriction; therefore the natural levels of glucose or lactate would be at baseline at the start of testing. The task was also chosen because it measures spatial working memory, which is sensitive to hippocampal manipulations. In the current experiments, animals were placed on a four-arm, plus-shaped maze (arms: 45 cm long, 14 cm wide, 7.5 cm tall; center area: 14×14 cm) constructed of opaque, black Plexiglas, as described previously [22], [23], [24] or in a four-arm, plus-shaped maze with slightly higher sides (45 cm long, 14 cm wide, and 15 cm tall; center area: 14×14 cm) that were made of clear Plexiglas. The maze with higher arms was used to contain the rats better on the maze in experiments in which a delay was imposed between arm choices [43], [44]. The maze was located in the center of the testing rooms on a table 76 cm above the floor surrounded by a rich assortment of extra-maze visual cues. During each testing session, the rat was placed in a start arm and allowed to explore the maze freely for 20 min while the number and sequence of arm entries were recorded. An alternation was defined as when the rat visited all four arms within a span of five choices. Thus, five consecutive arm choices during a testing session comprised a quintuple set. As examples, a quintuple set consisting of arm choices A,B,D,A,C was considered an alternation but a quintuple set consisting of arm choices A,B,D,A,D was not considered an alternation. Using this procedure, possible alternation sequences are equal to the number of arm entries minus 4. The percent alternation score is equal to the ratio of actual alternations/possible alternations ×100; chance performance using this measure is 44%.

Intrahippocampal injections of lactate were expected to enhance memory scores. To prevent a ceiling effect in memory scores, a delay of 20 sec was introduced between each arm entry. After the rat entered the first four arms, a barrier was placed at the end of the fifth choice to prevent the rat from leaving the arm for 20 sec. The first four choices made without the 20-second delays were not included in the calculations of spontaneous alternation scores.

All other pharmacological experiments examined impairments of memory with drugs that block lactate delivery from astrocytes to neurons, together with possible reversals of these impairments with co-administration of glucose or lactate. No delay between arm entries was imposed during these experiments or in the experiment measuring changes in extracellular glucose and lactate during maze testing.

The pharmacological manipulations were performed as within-subjects testing of multiple doses. Each testing session occurred in a new room with new extramaze visual cues to encourage sufficient exploratory behavior. Additionally, an interval of at least 48 hrs was imposed between testing sessions to allow sufficient time for drugs to clear the animal's system. To ensure an accurate assessment of spontaneous alternation, only rats that made a minimum of ten arm entries (6 possible alternations) during the 20-minute test were included in the final analysis.

### Bioprobe measurements of extracellular hippocampal glucose and lactate during behavioral testing

Either a glucose- or a lactate-sensitive biosensor was inserted into the dorsal hippocampus via a guide cannula (Pinnacle Technology Inc., Lawrence, KS). The biosensor projected 3 mm beyond the end of the implanted cannula. The last 1 mm of the probe was coated with lactate oxidase or glucose oxidase to metabolize the respective analyte, generating a current measured by the probe. The biosensor was connected to a potentiostat inside the head cap, which sent readings of the current generated by lactate or glucose in extracellular fluid to a computer by telemetry, with data recording and storage in 1-sec bins accomplished with Pinnacle Technology Laboratory v. 1.6.7 software. Glucose biosensors have previously been shown to have a range of 0–10 mM with *in vitro* sensitivity of 1.6±0.4 nA/mM (mean ± SEM) and lactate biosensors have a range of 0–8 mM with an *in vitro* sensitivity of 4.6±0.6 nA/mM (mean ± SEM) [45], [46]. To confirm the accuracy of the biosensors prior to implantation and immediately following testing, the probe was placed in 0.1 M PBS, connected to the potentiostat, and readings were allowed to stabilize (generally stable within 15–30 minutes). After a stable baseline reading over at least 4 minutes was recorded for the lactate probe, lactate was added to the PBS in 20 µM increments every 1.5 minutes to establish the nA/mM ratio. For the glucose probe, glucose was added to the PBS in 500 µM increments. Because the probes have been shown to measure ascorbic acid, they are coated with a selective membrane containing ascorbate oxidase to break down the ascorbic acid so it is not measured by the biosensor. To ensure this layer is intact, 250 µM of ascorbic acid was added to the PBS solution two times. No other substrates have been shown to be measured by the biosensors [45], [46]. Four rats were tested with the lactate biosensor and four rats were tested with the glucose biosensor. Biosensors were inserted into the guide cannula at least 4–5 hours prior to testing on spontaneous alternation. For graphical presentation in this report, the recordings are presented here as averages across 10 sec. Baseline values were determined using the 5 min prior to the start of spontaneous alternation and all results are reported as a percent change from baseline. The results were then analyzed statistically using comparisons at baseline, 0.5, 5 min, 10 min, and 15 min into spontaneous alternation testing as well as at 0, 0.5, and 5 min after the end of testing.

### Intrahippocampal Injections to enhance or impair memory

All injections (0.5 µl, 0.9% saline vehicle, pH = 7.2) were made bilaterally into the ventral hippocampus with a CMA/100 microinjection pump at a flow rate of 0.25 µl/min 5 min prior to behavioral testing. Ten rats were injected with lactate (0, 50, 100, or 150 nmol of lactate in 0.5 µl of 0.9% saline, pH = 7.2), 12 rats were injected with 1,4-dideoxy-1,4-imino-D-aribinitol (DAB; 0, 0.5, 5, 50 pmol, 50 pmol+25 nmol glucose, or 50 pmol+50 nmol lactate in 0.5 µl of 0.9% saline, pH = 7.2), and 8 rats were injected with α-cyano-4-hydroxycinnamate (4-CIN; 3, 10, 30 pmol, 30 pmol+25 nmol glucose, or 30 pmol+50 nmol lactate in 0.5 µl of 1% DMSO and 0.9% saline, pH = 7.2) were infused into the hippocampus 5 min prior to behavioral testing. DAB, an inhibitor of glycogen phosphorylase, was used to block glycogenolysis in astrocytes [32], [37], [47]. Low doses of 4-CIN were used to block the neuronal transporter, MCT2, while not blocking astrocytic MCT1 or MCT4 transporters [34], [35], [48], [49], [50], [51], [52], [53]. Doses were given in a counterbalanced order using a Latin squares design with each main treatment tested in separate groups of rats. In the DAB and 4-CIN experiments, the maximum doses (50 pmol of DAB or 30 pmol of 4-CIN) were co-administered with either 25 nmol of glucose or 50 nmol of lactate in a counterbalanced order after the dose-response curve had been defined to test whether impairments induced by DAB or 4-CIN could be reversed by either glucose or lactate. Because each glucose molecule yields two lactate molecules, the molar dose of lactate was twice that of glucose to match the stoichiometry [32], [54]. Because 25 nmol of glucose is similar to doses that have been demonstrated to enhance memory,15, 50 and 150 nmol of lactate were chosen to assess whether lactate could enhance memory to include an optimal dose (50 nmol) as well as a dose that is higher (150 nmol) and lower (15 nmol) [55], [56].

### Histology for glycogen localization and for cannula placements

On completion of testing, rats received an overdose of sodium pentobarbital followed by intracardial perfusion with ice-cold 4% paraformaldehyde in 0.1 M PBS. Brains were removed and postfixed overnight in 4% paraformaldehyde. They were then transferred to a 20% glycerol solution until they lost buoyancy (∼48 hrs). They were then sectioned on a cryostat (Leica CM1850, Leica Microsystems Inc., Germany) with 20 µm slices of prefrontal cortex, striatum, dorsal hippocampus, and ventral hippocampus saved for glycogen localization analysis using a periodic acid-Schiff's reagent method (PAS) previously demonstrated to highlight glycogen [57]. Prior to PAS staining, immunofluorescence methods were used to stain neurons with neuronal nuclear antigen (NeuN) and astrocytes with glial fibrillary acidic protein (GFAP) so that all sections were stained with PAS and consecutive sections were stained for NeuN and GFAP. Briefly, for GFAP immunofluorescence, the tissue was rinsed in 0.05 M PBS 4× for 10 minutes and then incubated in a 5% normal goat serum (NGS), 1% bovine serum albumin (BSA), and 0.2% Triton X-100 in 0.05 M PBS for 60 minutes to block endogenous proteins. Next, the tissue was transferred a solution containing 5% NGS, 1% BSA, 0.2% Triton X-100 and 1∶2000 dilution of Rabbit anti-GFAP (Millipore # AB5804) in 0.05 M PBS overnight at room temperature. Sections were then rinsed 4× in 0.05 M PBS for 10 minutes each. Finally the tissue was incubated for 2 hours in 1% NGS, 0.2% Triton X-100, and 1∶2000 dilution of Goat anti-rabbit Alexa Fluor 488 (Invitrogen #A11008). For NeuN immunofluorescence, the same rinsing and blocking procedures were followed. The tissue was then incubated in 5% NGS, 1% BSA, 0.2% Triton X-100 and 1∶2000 dilution of Milli-Mark FluoroPan Neuronal Marker ( Mouse IgG conjugated with Alexa 488; Millipore # MAB2300X) in 0.05 M PBS overnight at room temperature. All tissue was then mounted on silated slides and stained for glycogen. The PAS method used was previously described [57], [58]. Briefly, slides were oxidized in 0.5% periodic acid for 10 min at room temperature and then incubated in a saturated solution of dimedone in 80% ethanol for 1 hour at 60°C. After rinsing in distilled water slides were reacted in Lillie's cold Schiff's reagent for 30 minutes. Slides were next rinsed in running tap water for 5 minutes. Slides were then dehydrated, delipidated using Histoclear and coverslipped. Photomicrographs of the tissue were collected using a Leica CTR6000 microscope, a Leica DM600B camera, and Leica Application Suite (v. 3.7.0, Leica Microsystems Inc., Germany). The PAS stain fluoresces at an excitation wavelength around 525 nm and can be visualized using a red light (rhodamine) fluorescent filter [59], [60], [61]. Images were captured for each slice with the rhodamine and fitc (green) filters (for GFAP or NeuN). Images were then compiled using Photoshop v. 6.0 to look for colocalization.

Forty-µm sections were also taken from the same brains around the areas of cannula implantations to confirm placements using cresyl violet. Photomicrographs were taken using Image Pro Express (v. 5.1.0.12, Media Cybernetics, Inc., Bethesda, MD). No data had to be excluded from rats with either extensive tissue damage or from rats where the placement was outside the target brain structure; however one rat was excluded from the DAB experiment because it died prior to the completion of data collection. Two rats were excluded from the 4-CIN study; one developed seizures and the other removed one cannula prior to completion of data collection. Lastly, one rat implanted with a glucose biosensor was excluded because it failed to navigate the maze during the spontaneous alternation test.

To show that 4-CIN did not block monocarboxylate transport into mitochondria, activity of the mitochondria was monitored using a stain for succinate dehydrogenase (SDH) activity [62], [63]. SDH activity has previously been demonstrated to decrease in the presence of 3-bromopyruvate and with sensory deprivation [62], [63], [64] supporting a relationship between SDH activity and mitochondrial function. Prior to sacrifice, the rats used in the 4-CIN dose response experiment received 30 pmol 4-CIN unilaterally in the ventral hippocampus and 1% DMSO in saline in the other hemisphere, providing within-subjects comparisons. The hemispheres receiving 4-CIN were counterbalanced across rats. Five min after the injection animals were tested on spontaneous alternation for 20 minutes to parallel the other behavioral experiments. The rats then received an overdose of sodium pentobarbital and were perfused transcardially with 10% glycerol and 0.5% paraformaldehyde in PBS. The brains were removed, blocked, frozen rapidly in heptane cooled with dry ice, and sectioned in the coronal plane at 30 µm in a cryostat. Alternating 40 µm sections were saved from ventral hippocampus for cresyl violet nissl staining to determine cannulae placements and staining for SDH activity using methods previously described [62], [65]. The colorimetric change reflecting SDH activity was then assessed for optical density using ImageJ 1.43n (National Institutes of Health, USA, http://rsb.info.nih.gov/ij). In ventral hippocampus, separate measures were taken from CA1, CA3 and the dentate gyrus.

### Statistical Analysis

All statistical analyses were done using SPSS v. 18.0 (SPSS, Chicago, IL). For each experiment, repeated measures ANOVAs were used to analyze differences. Fisher's least significant difference post hoc tests were conducted when the results from the ANOVAs were significant.

## Results

### Glycogen is in astrocytes, not neurons

**Figure 2:**
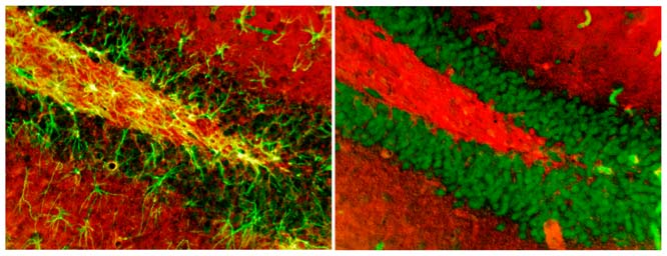
Immunolabeling of astrocytes using GFAP and staining for glycogen using a Periodic Acid Schiff's Reaction (PAS) demonstrated colocalization (in yellow) of glycogen and astrocytes (left). Immunolabeling of neurons using NeuN and glycogen with PAS showed no colocalization (in yellow) of glycogen in neurons (right).

As shown in Figure 2, astrocytes immunolabeled with GFAP (left) and neurons immunolabeled with NeuN (right) in the dentate gyrus are stained green. Glycogen colocalized with GFAP or NeuN is stained yellow. As evident in this figure, glycogen staining was colocalized with astrocytes but not with neurons. These findings support past results [66], [67], [68], [69]. The colocalization is especially prominent in the molecular layer and the hilus of the dentate gyrus, i.e. in synapse dense regions of the granule cell dendrites and the axonal regions where energy demands would be expected to be higher than in the cell layers [17], [70], [71]. Similar results (not shown here) were also seen in the prefrontal cortex, striatum, and ventral hippocampus with colocalization of glycogen and GFAP but no colocalization with NeuN.

### Lactate and glucose levels increase and decrease, respectively, during behavioral testing

**Figure 3:**
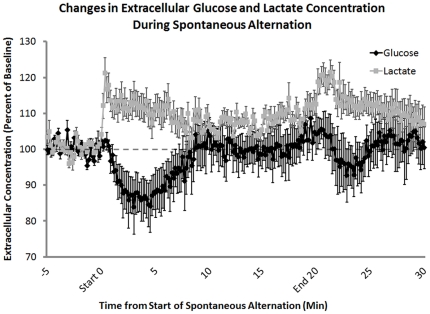
Extracellular lactate and glucose levels in the hippocampus, measured before, during, and after behavioral testing. Using lactate- and glucose-specific biosensors, extracellular concentrations of both lactate and glucose were measured during spontaneous alternation testing. Lactate concentrations significantly increased at the beginning of behavioral testing (n = 4; t_3_ = 4.77, p<0.02; MEAN ± SEM: 112.50%±3.15%). In contrast, glucose concentrations decreased after 5 minutes on the task (n = 3; t_2_ = 3.58, p<0.05; MEAN ± SEM: 86.19%±7.73%). The increase in extracellular glucose seen 5–10 min after the start of memory testing corresponds to an increase in blood glucose levels (baseline vs. 10 min: p = 0.47, 10 min MEAN ± SEM: 103.68%±6.29%). After the rat was removed from the maze there was a significant increase in lactate compared to baseline levels (t_3_ = 4.77, p<0.02; MEAN ± SEM: 117.9%±2.87%) most likely due to handling.

Recordings from bioprobes before, during and after memory testing revealed substantial increases in extracellular hippocampus levels of lactate and decreases in extracellular levels of glucose beginning with the placement of the rat on the maze (Figure 3). Extracellular lactate concentrations significantly increased in the first 30 sec of testing on the maze (t_3_ = 4.77, p<0.02) while glucose levels did not significantly decrease until 5 min into testing (t_2_ = 3.58, p<0.05). Both lactate and glucose levels returned toward baseline after about 10 min of testing, while the rats were still performing on the maze (baseline vs. 10 min: lactate, p = 0.12; glucose, p = 0.47) however at 15 minutes after the start of testing, lactate levels were significantly higher than baseline (t_3_ = 4.47, p<0.05). The initial decreases and later increases in extracellular glucose levels are consistent with those we have seen before with 5-min sampling obtained with microdialysis procedures in the hippocampus during similar spontaneous alternation testing; the increase in extracellular glucose late in testing corresponds to an increase in blood glucose levels during training [19], [21].

A slight second increase in lactate was evident when the rats were removed from the maze and replaced in their home cages (t_3_ = 3.27, p<0.05). This could indicate that the arousal of handling was sufficient to elicit these fluxes in lactate and supports previous findings that stress can elicit lactate production from glial cells [72]. However this effect at the end of testing was not as great or as long-lasting as was the rise in lactate during testing. It should also be noted that while no memory processing was measured behaviorally at the time of handling-related increases in lactate, it is likely that memory of the handling experience may also be formed in this experiment as in other experiments of this type.

### Intrahippocampal infusions of lactate enhance memory

**Figure 4:**
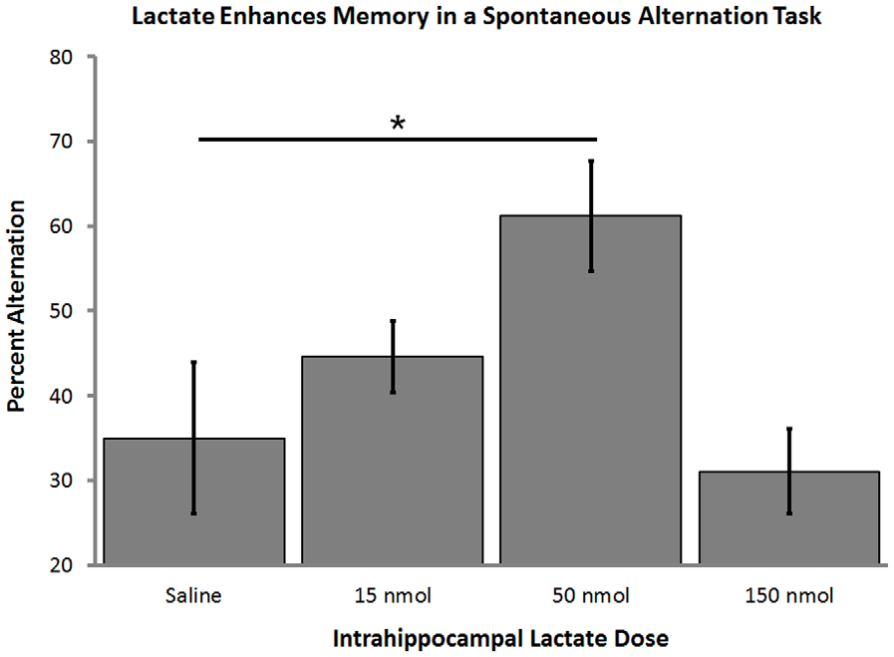
Enhancement of memory with intrahippocampal injections of lactate. Lactate injected into the ventral hippocampus 5 min before testing improved the percent alternation scores on a 4-arm delayed spontaneous alternation task at the 50 nmol dose (n = 10; F_3,27_ = 4.04, p<0.02; Percent Alternation ± SEM: Saline = 34.5%±8.9% vs. 50 nmol Lactate = 61.2%±6.5%). Higher and lower doses of lactate did not significantly improve alternation scores.

Injections of lactate directly into the hippocampus prior to testing enhanced memory on the delayed version of the spontaneous alternation task (F_3,27_ = 4.04, p<0.02; Figure 4). The enhancement of memory by lactate followed an inverted-U dose response curve in a manner similar to that seen previously with systemic and intrahippocampal injections of glucose [23], [55], [73]. In past studies of intrahippocampal injections of glucose, the optimal dose was ∼20 nmol [55]. This value is approximately half the optimal dose of lactate, consistent with two lactates produced for each glucose metabolized [20], [32], [54], [55]. There were no significant differences in the total arm choices the animals made across doses (p>0.9). There were also no differences across the counterbalanced testing sessions confirming there were no overall improvements or impairments due to repeated spontaneous alternation testing (p>0.3).

### Inhibition of glycogenolysis impairs memory, an effect reversed by addition of glucose or lactate

**Figure 5:**
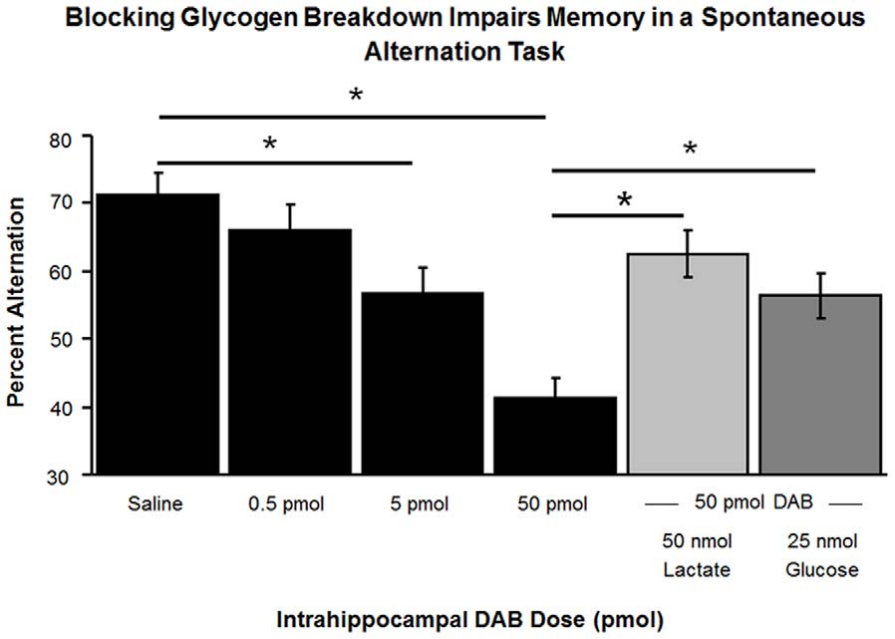
Impairment of memory by DAB injections, used to inhibit glycogenolysis. The impairment was reversed by lactate or glucose, which can act downstream of glycogenolysis. 1,4-dideoxy-1,4-imino-D-arabinitol (DAB) injected into the ventral hippocampus 5 min prior to testing significantly impaired scores on a 4-arm spontaneous alternation task (n = 12; Percent Alternation ± SEM: Saline = 71%±3.7% vs. 5 pmol DAB = 58.8%±3.6%, p<0.02 and vs. 50 pmol DAB = 41.6%±3.2%, p<0.001). The performance deficit created by 100 µM of DAB was significantly reversed by the co-administration of 100 mM lactate or 50 mM glucose (Percent Alternation ± SEM: 50 pmol DAB = 41.6%±3.2% vs. 25 nmol of glucose and 50 pmol DAB = 62.6%±3.1%, p<0.001 and 50 nmol of lactate and 50 pmol DAB = 56.2%±2.9%, p<0.01).

DAB was used to inhibit glycogen phosphorylase and to limit the production of lactate from glycogen in astrocytes. DAB injections into the hippocampus significantly impaired alternation scores (F_3,33_ = 15.48, p<0.001; Figure 5). The impairment was evident at both the 5 and the 50 pmol doses (Percent Alternation ± SEM: Saline = 71%±3.7% vs. 5 pmol DAB = 58.8%±3.6%, p<0.02 and vs. 50 pmol DAB = 41.6%±3.2%, p<0.001). The total number of arm choices did not differ across doses (p>0.6).

Injections of either 25 nmol of glucose or 50 nmol of lactate together with the higher DAB dose significantly reversed the memory impairments (Percent Alternation ± SEM: 50 pmol DAB = 41.6%±3.2% vs. 25 nmol of glucose and 50 pmol DAB = 62.6%±3.1%, p<0.001 and 50 nmol of lactate and 50 pmol DAB = 56.2%±2.9%, p<0.01).

### Blocking lactate transport into neurons impairs memory, an effect not reversed by addition of either lactate or glucose

**Figure 6:**
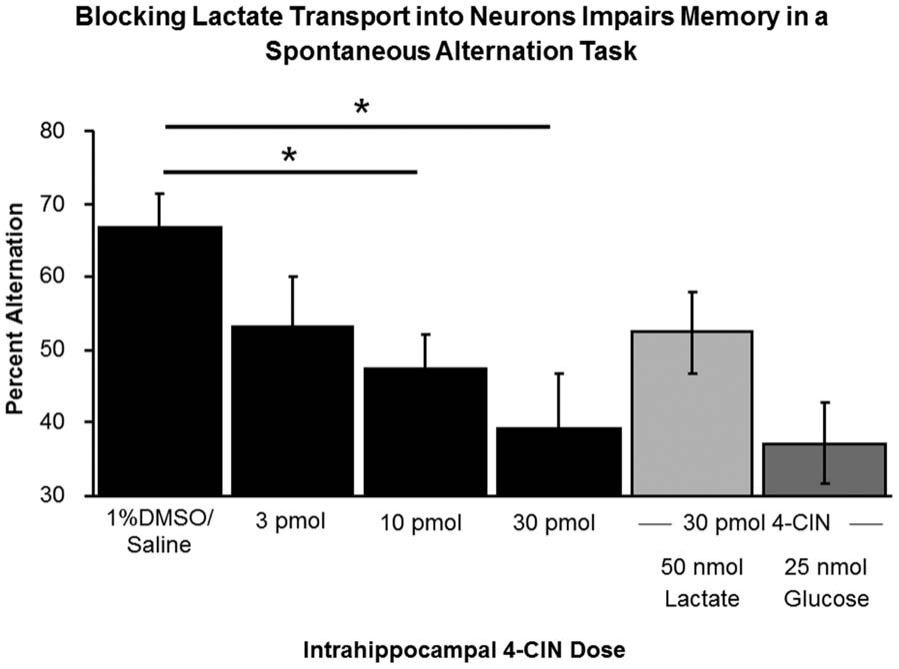
Impairment of memory by 4-CIN injections, used to block MCT2. The impairment was not reversed by either lactate or glucose. Blockade of the MCT-2 with 4-CIN impaired working memory in a dose-dependent manner (n = 6; Percent Alternation ± SEM: 1% DMSO in Saline = 65.2%±4.9% vs. 10 pmol 4-CIN = 47.4%±4.7%, p<0.05 and 30 pmol 4-CIN = 39.8%±3.3%, p<0.05). This impairment was not significantly reversed with the addition of either lactate or glucose (ps>0.1).

Preferential blockade of the MCT2 by intrahippocampal injections of 4-CIN significantly impaired alternation scores (F_3,15_ = 4.52, p<0.03; Figure 6). The impairment was seen at both the 10 pmol and the 30 pmol doses (Percent Alternation ± SEM: 1% DMSO in Saline = 65.2%±4.9% vs. 10 pmol 4-CIN = 47.4%±4.7%, p<0.05 and 30 pmol 4-CIN = 39.8%±3.3%, p<0.05). The total arm choices made did not differ across doses (p>0.9). Importantly, the addition of either 25 nmol of glucose or 50 nmol of lactate to the 30 pmol dose of 4-CIN did not significantly improve working memory (all p>0.1).

**Figure 7:**
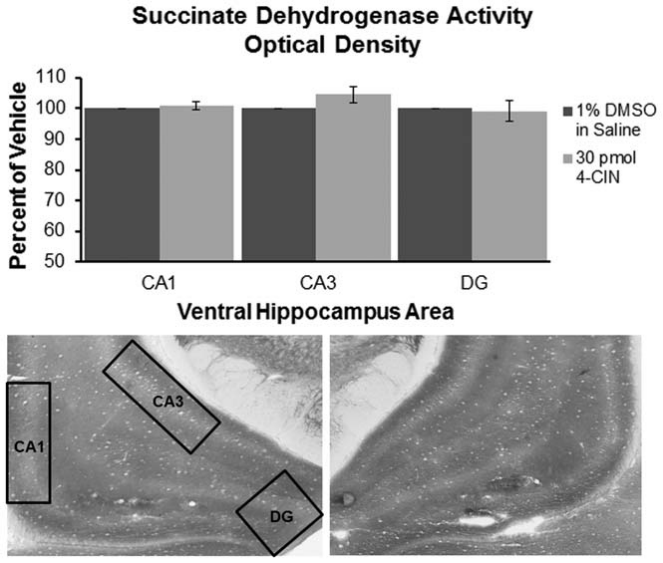
Representative histology showing SDH activity in the ventral hippocampus. In this example, the left hemisphere received infusions of 1% DMSO in saline (left) and the right hemisphere received infusions of 30 pmol 4-CIN. There were no significant differences in optical density between ventral hippocampal areas receiving 30 pmol of 4-CIN or 1% DMSO in saline in CA1, CA3, or dentate gyrus (n = 6; p>0.1).

In the ventral hippocampus, CA1, CA3 and DG were individually analyzed for optical density using Paxinos and Watson (2003) as a reference. The area of interest was highlighted and a minimal threshold was used to exclude portions that did not contain stained tissue (e.g. capillaries) and kept constant across animals. No significant differences in SDH activity were seen between the hemisphere that received 30 pmol of 4-CIN and the hemisphere that received 1% DMSO in saline across the assessed areas of the ventral hippocampus (Ns = 4; p>0.1, Figure 7).

## Discussion

The data reported here support the view that, in the hippocampus, glycogenolysis in astrocytes, and subsequent delivery of lactate to neurons, is important for spatial working memory. Within the hippocampus, glycogen was localized to astrocytes and not neurons, consistent with other evidence that astrocytes are the prime source of glycogen in the brain [28], [29], [69], [74], [75]. There are still some who argue glycogen is found in neurons as well, which could be due to the transient appearance of glycogen in neurons during development [76] or the appearance of glycogen in neurons in glycogen storage diseases [77], [78], [79]. Glycogen was further localized to the molecular layer and hilus, as compared to the dentate granule cell layer [80]; the synapse dense molecular layer is likely to be a subregion especially dependent on maintenance of energy metabolism to support neural functions [17], [81].

Using bioprobes to measure extracellular lactate and glucose levels in the hippocampus with 1-sec sampling, the present findings indicate that lactate levels increase, and glucose levels decrease, during alternation testing. The decrease in glucose levels is consistent with prior evidence obtained with slower 5-min *in vivo* microdialysis samples [18], [21]. Of note, the decrease in glucose levels in prior experiments was not a function simply of locomotor activity since the decrease was not seen on a similar alternation task with comparable motoric activity but lower cognitive demands [21]. Also, the rise in hippocampal glucose levels while the rats remain on the maze during testing corresponds to a rise in blood glucose levels, probably subsequent to epinephrine release from the adrenal medulla into blood and initiation of hepatic glycogenolysis, while the rats are tested [21].

The reciprocal relationship between lactate and glucose responses to memory testing suggests that glucose and lactate work in concert to maintain the energy capacity of neurons, as appears evident also in the dentate gyrus after perforant path stimulation [45] and in somatosensory cortex after whisker stimulation [33]. While it is clear that the increased levels of lactate are mirrored by decreased levels of glucose in the beginning of testing, the levels of extracellular lactate stay above baseline for the duration of spontaneous alternation testing even as glucose levels return to baseline values, suggesting that lactate may not just be a compensatory substrate but may be important for maintaining working memory processing.

The importance for memory of the increases in lactate was supported by a set of pharmacological findings showing that lactate provision from astrocytes to neurons is necessary for memory processing. Lactate itself, in the absence of other (impairing) treatment enhanced memory in the alternation task. These findings are similar to those observed previously with intrahippocampal glucose injections [8], [16], [20]. The inverted-U dose-response function for memory enhancement is typical of many cognitive enhancers, including glucose [3], [101], [102], [103], [104]; while this effect has been shown repeatedly, there is no consensus regarding its neurobiological bases. The glycogen phosphorylase inhibitor, DAB, impaired memory when injected into the hippocampus; the impairment was mitigated by either lactate or glucose. While the lactate most likely was directly taken up by the neurons, it was unclear from these data whether the glucose was being taken up by the neurons or the astrocytes in order to rescue memory after impairment by DAB. As illustrated in Figure 1, astrocytes could have provided lactate to neurons either through glycolysis or glycogenolysis. However, additional findings indicated that MCT2 blockade also impaired memory and that the impairment was not reversed by either glucose or lactate. The blockade of the MCT2 transporter should not affect the ability of neurons to admit glucose through the main neuronal glucose transporter GLUT3 [105], [106]. Thus, the current data suggest that lactate, provided by astrocytes via glycolysis or glycogenolysis, may be an important substrate for neurons during working memory by providing rapid additional energy at times of high need. As shown here, that need can be generated by cognitive demands.

The findings reported here are largely consistent with past examinations of lactate derived from astrocytes in regulating memory processing in chicks [107], [108], [109] and laboratory rodents [32]. In addition, memory after inhibitory avoidance training and long-term potentiation were impaired by interference with MCT1 and these impairments were reversed by lactate. In rats, training-related expression of molecular factors often associated with memory, pCREB and Arc, were also blocked after inhibition of MCT1 and these effects too were rescued by lactate [32]. Although the previous work focused on lactate contributions to consolidation of long-term memory, and included the suggestion that lactate was not necessary for short-term memory, the present findings reveal an important role for lactate in spatial working memory assessed with short-term tests, suggesting that the role of astrocytes and lactate include supporting short-term memory processing as well as the formation of long-term memories.

With the 1-sec sampling method used here to measure fluxes in extracellular lactate and glucose levels; it appears that the rise in extracellular lactate slightly precedes the decrease in glucose. This finding suggests that the astrocytic responses may anticipate energy needs rather than responding to them. In this regard, it is important to note that glycogenolysis in astrocytes can be initiated by activation of ß-adrenergic receptors on astrocytes [82], [83], [84]. Other neurotransmitters are likely involved as well, with evidence revealing several signals that can lead to glycogenolysis in astrocytes. These signals include neurotransmitters and modulators such as glutamate, GABA, vasoactive intestinal peptide, acetylcholine, serotonin, norepinephrine, dopamine, adenosine and insulin [85], [86], [87], [88], [89], [90], [91], [92], [93], [94], [95]. Some of these same modulators have also been shown to increase glucose transport into the astrocytes [96], [97], [98] and this increased glucose transport has been associated with learning and memory [99], [100]. These and other neurotransmitters have received attention in regulating memory processing, with interpretations based on direct neural actions of the transmitters and related drugs and other interventions. Given the breadth of neurochemical signals that act on astrocytic receptors, and possibly then on lactate production, it may be important to revisit the effects of many neurotransmitter-related treatments that enhance memory to determine which drugs act indirectly on neurons via regulation of astrocytes to provide energy substrates available for neurons.

Together, these findings suggest that astrocytes may play an important role in neural plasticity and memory [29], [31], [32], [96], [109], [110]. The neurochemical and pharmacological results are consistent with the hypothesis, illustrated in Figure 1, in which lactate is released from astrocytes and ‘shuttled’ to neurons for energy metabolism (as in [29], [111]). It must be noted that there is not uniform agreement about the role for lactate in providing energy for brain function [112]. In part, as presented in recent reviews [17], [112], the disagreement results from a dearth of information relating the magnitudes and time courses of fluxes in brain ECF glucose levels to those of ECF lactate levels under normal physiological conditions. The present findings address this issue directly with experiments showing that lactate levels increase when glucose levels decrease.

Although the focus of the present report is on metabolic contributions to modulation of memory, there are other ways that astrocytes may contribute to memory and other neural functions. These need not be seen as mutually exclusive and include: neurotransmitter clearance to inactivate neurotransmitters by uptake mechanisms; as one of the elements of the “tripartite synapse” [113], [114], with astrocyte, pre- and postsynaptic neural elements functioning in integrative manner to control excitability and to reshape synapse morphology (e.g., [115], [116], [117], [118], [119], [120]); synthesis and release of d-serine into extracellular space, where serine functions as an NMDA receptor co-agonist to promote long-term potentiation [121]; synthesis and release of ephrin-A3 in the hippocampus [122], which may regulate glial glutamate transport, and synapse morphology [118]. Together, there is growing evidence that astrocytes participate actively and importantly to memory processing and neural plasticity, requiring careful attention to the contributions of these varied astrocytic mechanisms for memory.
